# ADAPT study: adaptation of evidence-informed complex population health interventions for implementation and/or re-evaluation in new contexts: protocol for a Delphi consensus exercise to develop guidance

**DOI:** 10.1136/bmjopen-2020-038965

**Published:** 2020-07-20

**Authors:** Mhairi Campbell, Graham Moore, Rhiannon E Evans, Dmitry Khodyakov, Peter Craig, On behalf of the ADAPT Study team, Hannah Littlecott

**Affiliations:** 1 MRC/CSO Social and Public Health Sciences Unit, University of Glasgow College of Medical Veterinary and Life Sciences, Glasgow, UK; 2 The Centre for the Development and Evaluation of Complex Public Health Interventions, Cardiff University, Cardiff, South Glamorgan, UK; 3 Pardee RAND Graduate School, RAND Corp, Santa Monica, California, USA

**Keywords:** statistics & research methods, public health, health policy

## Abstract

**Introduction:**

Complex population health interventions that are effective in one context may not be effective elsewhere, and may even be harmful. As such, an intervention may require adaptation to ensure it fits with a new context. To date, there is no overarching guidance to help researchers to adapt and evaluate interventions in new contexts, and no criteria to support research funders or journals assess proposed or reported adaptations or evaluation. There is limited assistance for policy-makers and practitioners to decide if evidence-informed interventions are appropriate to their context, or if adaptation and further evaluation is needed. This Delphi exercise will contribute to the development of guidance for these communities to support the adaptation, implementation and/or re-evaluation of complex population health interventions in new contexts.

**Methods:**

We will conduct a Delphi consensus exercise to gather expert opinion from researchers, research funders, journal editors and policy-makers. Expert opinion will be sought on: appropriate definitions and concepts, identifying key methodological considerations and establishing adaptations and processes to be undertaken during adaptation of complex population health interventions in new contexts.

**Ethics and dissemination:**

Ethics approval for the Delphi exercise has been obtained from the University of Glasgow and and the RAND institutional research board. Dissemination of the results of this study will be through peer-reviewed publications, workshops at national and international conferences, and a summary of the guidance developed for key organisations and stakeholders.

**Strengths and limitations of this study**

- This study will use the best practice methods to gather opinion from international experts on the adaptation of population health interventions.
- The results of the Delphi exercise will contribute to the development of guidance for practitioners, policy-makers, researchers and funders on the adaptation of complex population health interventions for implementation and/or re-evaluation in new contexts.
- Guidance on the adaptation of complex population health interventions will help prevent resources from being wasted on inappropriate adaptations and improve understanding of how best to evaluate adapted interventions.

## Introduction

Health service and public health programmes and policies to improve population-level health often involve multiple interacting components, and produce effects through interaction with the contexts in which they are implemented.^1–3^ Producers and users of evidence often wish to take population health interventions that have been shown to be effective in one context and implement them in other contexts. Context can be broadly defined as circumstances in which the intervention occurs that may interact with the intervention for example, the geographical setting, population, cultural or regulatory conditions.^4 5^ However, an intervention that works well in one context may not be deliverable or effective elsewhere or may cause harm.^6^ It is often necessary to evaluate whether the intervention and its effects can be replicated, or whether and to what extent the intervention requires to be adapted to fit the new context.^1^ In addition, there is a lack of conceptual development in research on context and adaptation of complex population health interventions, and the possibility of key misunderstandings within the topic of adaptation.^5^

Currently, there is no overarching guidance for adaptation of complex population health interventions for implementation and evaluation in other contexts.^5^ Such guidance should help practitioners and policy-makers to decide whether complex population health interventions are appropriate for their context, support researchers to adapt interventions for new contexts, and help research funders to decide on the case for supporting adaptation studies. It should, therefore, help producers and users of evidence to avoid wasting resources on inappropriate adaptations of interventions that are unlikely to be effective, or on large-scale evaluations of interventions in new contexts when there is little likelihood of effectiveness.

The ADAPT study aims to provide guidance to practitioners, policy-makers, researchers and funders to support the adaptation of complex population health interventions for implementation and/or re-evaluation in new contexts. The resulting guidance from this study will complement existing guidance on conducting complex interventions,^1^ process evaluation^7^ and developing complex interventions.^8^ This protocol outlines a Delphi consensus exercise, the third of four work packages in the ADAPT study. The first work package of the ADAPT study involved a systematic review of current guidance,^5^ which has identified themes for further investigation within both ongoing qualitative work (the second work package), and a Delphi consensus exercise and consensus meeting (third work package). The fourth work package oversees integration across work packages and dissemination of the finalised guidance. The rationale and overarching objectives of the overall ADAPT study are outlined in Evans *et al*.^9^ We will use a Delphi consensus exercise to solicit input from a large and diverse group of leading researchers, international-level representatives from research funders and academic journals and national-level policy-makers, and explore the existence of consensus among them. We shall develop guidance based on the work packages of the ADAPT Study, which will be finalised after review at a consensus workshop.

### Aim

The Delphi consensus exercise will gather expert opinion from researchers, research funders, journal editors and policy-makers to address the following questions:

What concepts, definitions and nomenclature should be used when conducting and reporting on the adaptation of complex population health interventions in new contexts?

What are the key adaptations to be considered when implementing complex population health interventions in new contexts (eg, adapting the intervention, implementation procedures and/or context)?

What processes should be undertaken when adapting complex population health interventions in a new context?

What criteria should inform the extent of intervention re-evaluation to be undertaken in the new context?

What are the most important methodological considerations when adapting, implementing and/or re-evaluating complex population health interventions in new contexts?

How consistent are views on best practice between researchers, research funders, journal editors and policy-makers?

## Methods

### Advisory group

The ADAPT study has an advisory group comprising leading academic, policy and practitioner experts in the field of complex population health interventions, including representatives of funding bodies. The role of the advisory group is to provide expert advice to the project. The advisory group will be invited to participate in the Delphi consensus exercise and contribute to the consensus meeting.

### Development of Delphi consensus exercise

To develop the Delphi exercise, we will follow recommendations for the best practice in developing reporting guidance.^10^ We will conduct a modified Delphi consensus exercise, the standard method for obtaining expert opinion.^11 12^ To do this, we will use the RAND ’s ExpertLens system for expert elicitation and stakeholder engagement.^13 14^ The method involves a modified Delphi structure with invited experts providing feedback on potential guidance, followed by repeated iterations of the potential guidance.^13^ The ExpertLens process includes an online discussion of the guidance content among the participating experts. There will be three rounds of online exercises (two surveys and a discussion round), which will be hosted electronically and will have an international reach. The consensus meeting will be hosted in person in the UK, with opportunities for international experts to join remotely.

The initial content for the Delphi consensus exercise is informed by Work Packages 1 (systematic review of existing guidance) and 2 (qualitative work with researchers and other stakeholders involved in adaptation studies) of the ADAPT study.

Work Package 1 aims to understand how adaptations of interventions are conducted, how adaptations to new contexts are explained, and how any existing guidance on the adaptation of interventions is used. To facilitate these aims, Work Package 1 comprises a systematic review of existing guidance, and a scoping review of examples of complex population health interventions that have been adapted for different contexts. The protocol for the systematic review is available,^2^ and the final review is published in Implementation Science.^5^ The scoping review protocol is available on the Open Science Framework, and the publication is in preparation.

Work Package 2 aims to understand how intervention adaptation decisions are made and justified, the procedures followed and the use of guidance. Work Package 2 consisted of semistructured interviews with researchers, representatives of international-level research funding bodies, peer-reviewers, practitioners and policy-makers with experience of delivering, evaluating or commissioning population health interventions adapted for a new context. Authors of academic articles of intervention adaption were identified from the Work Package 1 systematic review and invited to participate in the Work Package 2 interviews. Relevant stakeholders in the research community were also recruited for Work Package 2, representing funders from international funding boards, journal editors and peer-reviewers of journals reporting on intervention adaptations or examples of adapted and/or re-evaluated interventions. The respondents for Work Package 2 were identified using a sampling framework for a case study research design of a range of types of population health interventions and evaluation outcomes. The interviews examined: how intervention adaptation, implementation or re-evaluation in new contexts is undertaken; how this is justified and assessed; and how successes or failures in replicating intervention effects are attributed. At the time of writing this protocol for the Delphi component of ADAPT, analyses and write up of qualitative data are in progress.

The results from Work Packages 1 and 2 will be used to identify key issues for Delphi participant consideration. The issues will cover the following categories:

1. Concepts, definitions and nomenclature.
2. Types of adaptation to be considered.
3. Procedures for adaptation.
4. Criteria to inform the extent of intervention evaluation to be undertaken.
5. Important methodological considerations.

The ExpertLens process will involve developing survey questions, asking respondents to rate and explain the importance of each issue, how it might be refined, how it might be addressed and any issues that have been missed. The opening page will set out the aims of the Delphi consensus exercise, key concepts and terms, predicted time of completion, deadline for responding, data management and reporting. Cognitive question testing will be used with a small group of researchers (n≤5) outside of the study team to finalise format and item wording. Ethical approval for this work package of the ADAPT study has been obtained from the University of Glasgow College of Social Sciences Ethics Committee (reference number 400190054). Ethical approval has also been obtained from the RAND Institutional Research Board where the consensus exercise is being distributed (reference number 2019–0937).

### Participants

We will invite around 100 key international experts identified from the reviews in Work Package 1, participants from Work Package 2 interviews, plus additional representatives from researchers, research funders, journal editors and policy-makers identified by study coapplicants. Knowledge within the ADAPT project team and project management group will be used to add to the list of people with professional expertise of adaptation or re-evaluation of complex population health interventions. Respondents will be internationally located. We will aim to achieve a sample of 50 participants.

### Recruitment

Potential participants will be contacted via their publicly available work email addresses and invited to participate in the Delphi consensus exercise. The work email addresses will be collected from published journal articles and organisational websites. The email will outline the purpose of the project and provide a weblink to the online consensus exercise. The introductory page of the exercise provides a clear plain language statement explaining the consensus process. The introductory page of the exercise, prior to starting the exercise, includes a consent clause highlighting that by clicking the ‘accept’ button, the participant is consenting to take part in the consensus exercise. The exercise will be conducted entirely online. To require participants to provide written consent before completing the exercise would be overly onerous on their part. The invitation email, and the introduction to the exercise, will state clearly that the participants are not required to complete the online exercise and that the participant can stop at any stage.

One week prior to the start of round 1, members of the ADAPT team will send an email to individuals, introducing the ADAPT study and providing a participant information sheet. An email will be sent 1 day prior to the start of round 1, inviting individuals to participate in the consensus exercise. Up to three email reminders will be sent to non-responders. If response rates are low, up to two of the reminders will be sent from a member of the research team.

The confidentiality of participants will be protected, with responses and discussion comments identified by an allocated username. The consensus exercise data will be stored according to University of Cardiff guidelines for 10 years following completion of the study, and then destroyed, and information provided by participants will be dealt with in accordance with RAND’s ExpertLens Data Safeguarding Plan. All links between participants’ email addresses and their panel responses will be destroyed by the RAND at the end of the study.

### Structure of the Delphi consensus exercise

The consensus exercise will consist of three rounds: two rating surveys (rounds 1 and 3); and a discussion and feedback panel between rounds 1 and 3 (round 2).

#### Round 1

Questions for round 1 will be developed based on findings from Work Package 1 systematic review and scoping review, and Work Package 2 interviews. Participants will be invited to: rate their agreement with the clarity and usefulness of terminology relating to adaptation of interventions; rate their agreement with the description of characteristics of adaptation of interventions; and rate the importance of processes involved in exploring, developing, delivering and evaluating adaptation of interventions. Each question is presented on a scale from 1 to 9, for example, 1 ‘not at all important’ to 9 ‘very important’, to facilitate analysis of the responses. All items will include space for free-text comments, in which participants will be encouraged to explain their rating.

Round 1 will be available to complete for 1 week in the first instance, if necessary this will be extended for a further week. The quantitative responses to round 1 questions will be automatically analysed, calculating mean, median and quartiles, and assessing whether or not consensus has been achieved using the RAND/UCLA Appropriateness Method (RAM).^13 15 16^ The qualitative data will be collated using the framework developed in Work Package 1. See 'Analysis of consensus exercise data section' below for further details on data analysis.

#### Round 2

Participants will be presented with the median and IQR scores for each question, how their own response compared with the group, and whether consensus was achieved. The participants will be invited to participate in an anonymous online discussion of their responses. The aim is for participants to share their perspectives on the responses and issues relating to the round 1 questions. Members of the ADAPT study team will act as moderators for the discussions, following guidance on how to moderate the online discussion.^13 17^ If there is little engagement, or discussion is only on one or two issues, we will prompt discussion with unbiased, open-ended questions. The discussion panel is conducted via the ExpertLens software. Researchers and other participants will not be able to identify participants’ names.

#### Round 3

Participants will be presented with the results of round 1 and round 2 discussions. Each participant will be able to see the collated results of all participants and their own responses to each round 1 item. Participants will be invited to rate the items again, with the option of ‘Use my answer from Round 1’ if their round 1 response has not changed for an item, and encouraged to use the text box to explain why their answer has or has not changed.

### Analysis of consensus exercise data

We will use descriptive statistics to present quantitative data from rounds 1 and 3, and qualitative data from free-text responses will be analysed using the framework developed in Work Package 1. Descriptive statistics will consist of percentage response rates for each rating option, and median and quartile rates of responses. For the framework analysis, data will be coded according to a priori themes, and populated into a framework matrix. The framework developed for Work Packages 1 and 2 will inform the themes for the Delphi analysis framework. Continuity of the framework themes will facilitate direct comparison of current guidance, current practice and expert opinion. Consensus for each item or statement in the exercise is calculated using the RAM approach.^15^ Briefly, consensus involves determining whether the distribution of responses is bimodal and then identifying which tertile the median fell into (eg, scores of 1–3, 4–6 or 7–9 on the 9-point scale).^15 16^ Mean absolute deviation from the median will be used to identify disagreement about an item, with reduction of the mean absolute deviation from the median used to show that consensus is increasing.

### Draft development and consensus building meeting

The ADAPT study team will draft guidance, informed by the results of the three-round consensus exercise, the earlier work packages. Participants from the Delphi process will be invited to a full-day consensus meeting to review the guidance. We will aim to recruit a maximum of 25 participants from the project advisory group and participants of the Delphi consensus exercise, including policy-makers, representatives of funding boards and academic researchers, to achieve a diverse group of delegates. We will progress through each recommendation in the draft, focusing on areas of agreement and disagreement. Final recommendations for inclusion in the guidance will be agreed through discussion.

Participants of the consensus meeting (as well as invitees who are unable to attend the meeting) will be given the opportunity to provide written comments on these recommendations before coapplicants issue the final guidance, which will be published in a peer-reviewed journal and in extended format on the ADAPT study webpage. In addition to the main guidance, we will develop accessible summaries that are tailored to the different target communities, for example, academic researchers, policy-makers, practitioners, in order to support its uptake.

### Patient and public involvement

No patient involved.

## Ethics and dissemination

The Delphi consensus exercise has ethical approval from the University of Glasgow College of Social Sciences ethics committee (reference number 400190054) and the RAND institutional research board (reference number 2019–0937).

Dissemination of the results will be through peer-reviewed publications, presentations, symposiums and workshops at key national and international academic conferences, and a summary of the guidance will be disseminated to key organisations and stakeholders including policy-makers and practitioners.

## Supplementary Material

Reviewer comments
