# Supplementary material for: ADAPT study: adaptation of evidence-informed complex population health interventions for implementation and/or re-evaluation in new contexts: protocol for a Delphi consensus exercise to develop guidance
Source: BMJ Open. 2020 Jul 20;10(7):e038965. doi: 10.1136/bmjopen-2020-038965 (PMC7375505; doi:10.1136/bmjopen-2020-038965)
Supplement: Reviewer comments [file bmjopen-2020-038965.reviewer_comments.pdf]

PEER REVIEW HISTORY

BMJ Open publishes all reviews undertaken for accepted manuscripts. Reviewers are asked to complete a checklist review form (**http://bmjopen.bmj.com/site/about/resources/checklist.pdf**) and are provided with free text boxes to elaborate on their assessment. These free text comments are reproduced below.

ARTICLE DETAILS

**TITLE (PROVISIONAL)**	The Adapt Study: Adaptation of evidence-informed complex population health interventions for implementation and/or re-evaluation in new contexts. Protocol for a Delphi consensus exercise to develop guidance
**AUTHORS**	Campbell, Mhairi; Moore, Graham; Evans, Rhiannon; Khodyakov, Dmitry; Craig, Peter

This article was not externally reviewed at BMJ Open. Protocols that have been independently assessed prior to submission to BMJ Open are usually fast-tracked to publication on the grounds that further substantial changes will not be possible. This independent assessment will usually be external, independent review for both a substantial grant award from a non-commercial or government funder and ethics approval.
